# The Therapeutic Strategy of HDAC6 Inhibitors in Lymphoproliferative Disease

**DOI:** 10.3390/ijms19082337

**Published:** 2018-08-09

**Authors:** Maria Cosenza, Samantha Pozzi

**Affiliations:** Targeted Therapies in Oncohematology and Osteoncology, Department of Diagnostic Clinical and Public Health Medicine, University of Modena and Reggio Emilia, 41124 Modena, Italy; maria.cosenza@unimore.it

**Keywords:** histone deacetylase, histone deacetylase inhibitor, HDAC6, HDAC6 inhibitors, lymphoproliferative disease, epigenetic

## Abstract

Histone deacetylases (HDACs) are master regulators of chromatin remodeling, acting as epigenetic regulators of gene expression. In the last decade, inhibition of HDACs has become a target for specific epigenetic modifications related to cancer development. Overexpression of HDAC has been observed in several hematologic malignancies. Therefore, the observation that HDACs might play a role in various hematologic malignancies has brought to the development of HDAC inhibitors as potential antitumor agents. Recently, the class IIb, HDAC6, has emerged as one potential selective HDACi. This isoenzyme represents an important pharmacological target for selective inhibition. Its selectivity may reduce the toxicity related to the off-target effects of pan-HDAC inhibitors. HDAC6 has also been studied in cancer especially for its ability to coordinate a variety of cellular processes that are important for cancer pathogenesis. HDAC6 has been reported to be overexpressed in lymphoid cells and its inhibition has demonstrated activity in preclinical and clinical study of lymphoproliferative disease. Various studies of HDAC6 inhibitors alone and in combination with other agents provide strong scientific rationale for the evaluation of these new agents in the clinical setting of hematological malignancies. In this review, we describe the HDACs, their inhibitors, and the recent advances of HDAC6 inhibitors, their mechanisms of action and role in lymphoproliferative disorders.

## 1. Introduction

For a long time, cancer has been considered the result of a series of DNA mutations that induce the activation of oncogenes and the inactivation of tumor suppressor genes.

In addition to the modifications of the gene sequence, the epigenetic alterations of DNA also play a crucial role in the development of the tumor, deregulating gene transcription and contributing to the neoplastic process. Tumorigenesis is therefore the result of both genetic and epigenetic events [1]. “Epigenetic” is defined as biochemical changes of DNA chromatin that control the tertiary DNA structure resulting in modification of DNA gene expression that are not mediated by modifications in the primary nucleotide sequence. Epigenetic changes modify chromatin structure and the accessibility of DNA, thus regulating gene expression without altering the primary DNA sequence. It has been linked to the development of malignant phenotype and tumor progression, as a possible result of anomalous silencing of multiple tumor suppressor genes [2,3,4]. This process includes DNA methylation, phosphorylation, and histone acetylation that alter DNA accessibility and chromatin structure without modifications in the DNA sequence. Histone acetylation and deacetylation are regulatory mechanisms that play an important role in the control of gene transcription, affecting the interaction between DNA and histones. Histone acetylation induces activation of gene transcription, whereas deacetylation is associated with transcriptional repression. The two processes are catalyzed by two specific enzymes, histone acetyltransferases (HAT), and histone deacetylases (HDACs) [5]. HAT acetylate either arginine-(R) or lysine-(K) residues. Acetylated chromatin has a relaxed structure that promote gene transcription. HDACs are regulators of gene expression that enzymatically remove the acetyl group from arginine and lysine residues in histones [6]. HDACs stabilize and condense chromosome, making it less available for transcription factors. Interruption of HAT and HDAC activities has been related with the development of a variety of human cancers including hematological malignancies [7]. Interference with HDACs activity may influence both gene expression and other cellular processes including cell apoptosis, growth arrest, differentiation, and inhibition of angiogenesis.

HAT and HDAC not only affect histones but modify a large variety of non-histone proteins whose activity depends on their acetylation status, such as transcription factors, signal transduction mediators, structural proteins, and inflammation mediators [8,9]. The non-histone proteins, regulated by HDAC, work in nucleus, cytoplasm, and mitochondria and are involved in different pathways associated in cancer including lymphoproliferative disease [8,9].

In this review, we describe the HDACs, their inhibitors and in particular the recent advances of the selective histone deacetylases six inhibitors, their mechanisms of action and their role in lymphoproliferative disorders.

## 2. HDAC Classification and Their Physiological Roles

### 2.1. HDAC Classification and Their Physiological Roles in Lymphoid Lineage

HDACs differ in their structure, substrate specificity, enzymatic mechanism, subcellular localization, and tissue-specific expression. HDACs comprise a family of 18 enzymes, grouped in four classes, that play diverse roles in mammalian cell homeostasis and in tumor growth. The four classes are based on their sequence homology to their yeast orthologues: (1) Class-I HDACs includes HDACs 1, 2, 3, and 8, they are widely expressed in the tissues, located in the nucleus, and are involved in cell proliferation and survival; (2) class II family HDACs seem to have tissue-specific roles depending on the phosphorylation status. They can shuttle between the cytosol and nucleus. They are divided into two subgroups, class-IIa that comprises HDACs 4, 5, 7, and 9 and class-IIb, located in the cytoplasm and nucleus; includes HDACs 6 and 10. HDAC6 alone is specific for alpha tubulin, an important protein required for cell mitosis and movement; (3) class III, known as sirtuins 1–7, require nicotinamide adenine dinucleotide (NAD^+^) as a coenzyme for their activity; (4) class IV, which exhibits features of class I and II includes only HDAC 11 localized in the nucleus and has been implicated in the regulation of interleukin-10 expression [10] (Table 1).

HDACs are involved in diverse pathways and functions in the cells; they often occur in complexes and are involved in a network of interactions. In physiologic condition, all HDACs, with the exception of HDAC8, mediate their functions through complex macromolecular formation that very often include more than one HDAC and corepressor proteins such as Sin3A, N-Cor (nuclear receptor co-repressor), and SMRT (silencing mediator of retinoid and thyroid receptors).

In particular, HDAC1 and HDAC2 interact with each other to form the catalytic nucleus of multiproteotic complexes including the Sin3A, NURD (nucleosome remodeling and deacetylation) and Co-Rest complexes (co-repressor for element-1-silencing transcription factor). In the mammalian cell nucleus the HDAC1 and HDAC2 are predominant, and the concomitant deletion of HDAC1 and HDAC2 in T-cells [11] and ES (embryonic stem) cells induces a 50% decrease of total HDAC activity. HDAC3 has generally been found associated with complexes with N-Cor and SMRT proteins [12]. HDAC6, on the other hand, has works in macro-complexes that are involved in the ubiquitin pathway [13]. HDACs belonging to different classes can then coexist in the same complexes and adjust their activity to each other (HDAC3 with either HDAC4 or HDAC7) [14,15]. Previous studies demonstrated that individual HDAC members also manage the development and function of specific T cell lineages. HDAC1 suppresses Th2 cytokine production in airway inflammation [16]. HDAC3 is required for the development of both iNKT (invariant Natural Killer T cell) cells and CD8+ memory T cells [17].

Although the role of HDAC4 in T cell lineages remained unclear, a series of studies has established a potential link between HDAC4 and immune regulation. The expressions of multiple immune-related transcription factors, including c-Jun [18], NF-κB, and Bcl-6 [19,20], are controlled by HDAC4. One study conducted by Liu et al. reported that HDAC4 is differentially expressed in conventional T cells and iNKT cells residing in various lymphoid organs. HDAC4 deletion in T cells did not affect T cell development, maturation, or cytokine-secreting function [21].

The export of HDAC7 from the nucleus, which is necessary for the negative and positive selection of the thymocytes, affects the expressions of adhesion molecules and cytokines together with their receptors, that regulate the activity of cytotoxic T lymphocytes (CTL) [22,23]. Studies conducted by Azagra et al. [24] explored the potential role of HDAC7 in B cell development by generating a conditional knockout mouse model. The deletion of HDAC7 induces lymphopenia in peripheral organs secondary to the arrest of early B cell development. HDAC7 suppress myeloid and T lymphocyte genes in progenitors of the B lineage, interacting with myocyte enhancer factor 2C (MEFC2). In B cell progenitors, HDAC7 is recruited to promoters and enhancers of target genes. When HDAC7 is absent there is increased histone active marks [24]. HDAC6, HDAC9, and Sirt1 are able to mediate the histone deacetylation of the *Foxp3* gene, thus regulating Treg cell functions [25,26].

As previously reported, the activity of HDACs is regulated by different mechanisms such as for example, post-translational modifications (acetylation and phosphorylation) and interactions between proteins or the availability of cofactors essential for their enzymatic activity [27,28]. The cellular and physiological functions of acetylation are not limited to the regulation of gene expression. The acetylation assumes a wider significance in many physiological processes, as it also targets non-histone proteins as transcription factors, enzymes that repair DNA, chaperone proteins, and structural proteins.

The activity of many intracellular proteins is regulated according to their acetylation [27] and HDACs appear to be involved in a plethora of important cellular process comprising cell proliferation, cell migration, angiogenesis, and protein–protein interaction [27]; as in the case of the transcription factor STAT3. Through cytokine stimulation, STAT3 is activated and acetylated before homodimerization and translocation into the nucleus. HDAC-mediated deacetylation prevents dimerization and subsequent translocation into the nucleus [29,30].

The deacetylation of the HIF1 factor, expressed in the cell in response to available oxygen changes and angiogenesis, instead, prevents the association with the VHL complex, its ubiquitination, and the consequent degradation in the proteasome [31].

### 2.2. Biological Roles of Histone Deacetylase 6 (HDAC6)

**Figure 1:**
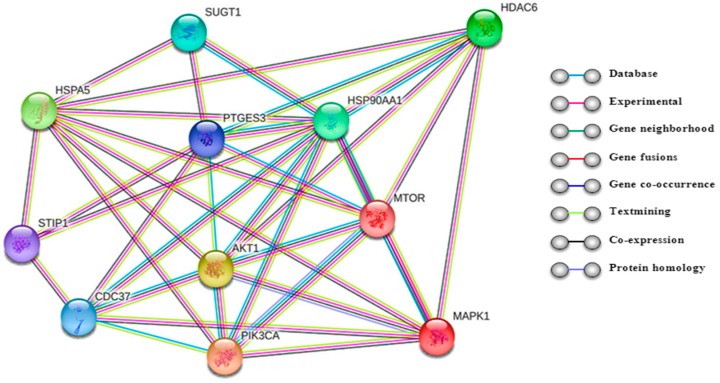
HDAC6 directs a variety of cellular processes that are important for cancer pathogenesis. Genetic interaction network using String (available online: https://string-db.org, accessed on 5 July 2018) that evaluates pathways and visualizes the connection among target genes according to the literatures search. (**HDAC6**: histone deacetylase 6. **HSP90AA1**: heat shock protein 90 kDa alpha (cytosolic). **MAPK1**: mitogen-activated protein kinase 1 serine/threonine kinase. **PIK3CA**: phosphoinositide-3-kinase (PI3K). **AKT1**: serine/threonine-protein kinases. **MTOR**: serine/threonine kinase. **HSPA5**: Heat shock 70 kDa protein 5 (glucose-regulated protein, 78 kDa). **SUGT1**: ubiquitination and subsequent proteasomal degradation of target proteins. **CDC37**: Co-chaperone that binds to numerous kinases and promotes their interaction with the Hsp90 complex. **STIP1**: stress-induced-phosphoprotein 1).

HDAC6 has also been studied in cancers especially for its ability to coordinate a variety of cellular processes that are important for cancer pathogenesis [32] (Figure 1). HDAC6 (class IIb) is one isoform of a family of HDACs enzymes that catalyzes the removal of functional acetyl groups from proteins. It is mainly localized in the cytoplasm and has been described as a tubulin deacetylase that has effects on microtubule-mediated processes through both deacetylase-dependent and independent mechanisms [33,34]. HDAC6 itself exerts both enzymatic and non-enzymatic actions on cell function. The growing interest for HDAC6-selective inhibitors is related to the modulation of acetylation of non-histone regulatory proteins (α-tubulin) implicated in cancer initiation and progression. Previous studies have focused on how the deacetylation of tubulin affects cell migration, metastasis, angiogenesis, and stress–response pathways [35,36].

Another possible role of HDACs in the development of hematological malignancies is related to the functional network of HDAC6 and HSP90. Hsp90, chaperone heat shock protein, was the second HDAC6 substrate identified in the cytoplasm after α-tubulin [37]. This protein is expressed in response to cellular stress, acting on client proteins involved in proliferative and antiapoptotic signaling and cell cycle control. HDAC6 modulates the chaperone activity of HSP90 through its deacetylation and, indirectly can regulate the stability of the HSP90 which controls the stability of many oncoproteins [38]. Chaperone Hsp90 has been defined as components of the IKK complex which is the core element of the NF-κB cascade and is involved in propagating the cellular response to inflammation.

IKK complex connected with its co-chaperone cdc37 works as a stabilizing factor of IKK through interaction between cdc37 and the kinase domains of IKKα and IKKβ in NF-κβ signaling [39]. HDAC6 works as a regulator of the ubiquitin and proteasome system (UPS) and therefore of the cellular response to protein misfolding [40,41,42]. HDAC6 is an element of the aggresome, a cellular structure that constitutes the major site of degradation for misfolded protein aggregates, both non-ubiquitinated and ubiquitinated misfolded proteins. The aggresome is an alternative pathway to the proteasome for the elimination of misfolded protein accumulation [43]. Transformed cells accumulate more misfolded proteins which are disposed by the proteasome and the aggresome [43,44]. Direction of misfolded proteins to the aggresome is essential for cell survival, since these proteins are form cytotoxic aggregates that can interfere with normal cell function. HDAC6 binds both polyubiquitinated misfolded proteins and dynein motors and act recruiting misfolded protein cargo to dynein motors for transport to aggresomes. The polyubiquitinated misfolded proteins are transported by microtubules to an autophagosome, where they are degraded via autophagy. This pathway is vital to multiple myeloma (MM) cells that overproduce misfolded proteins and overburden the proteasome degradation pathway [40,45]. Targeting both proteasomal and aggresomal protein degradation systems with proteasome and HDAC6 inhibitors, respectively, induces accumulation of polyubiquitinated proteins, activating the apoptotic cascades and synergistic cytotoxicity [42,46]. HDAC6 acts on GRP78 (78 kDa glucose-regulated protein), part of the unfolded protein response (UPR), inducing its deacetylation. As consequence of the acetylation, GRP78 dissociates from PERK (protein kinase RNA-like ER kinase) activating UPR and resulting in cell death [47]. HDAC6 is also involved in autophagy through the deacetylation of autophagy linked proteins. HDAC6 acts deacetylating LC3B-II (microtubule-associated protein 1 light chain 3), a central regulator of autophagy, involved in the degradation of p62/SQSTM (sequestrome 1) [48].

HDAC6 is a critical regulator of the pro-apoptotic p53. Recent studies of the selective HDAC6 inhibitor A452 tested in colorectal cancer cell lines, demonstrated to affect p53 and HSP-90, increasing the level of wild-type p53 as a result of the destabilization of MDM2, but decreasing mutant p53 and the consequent inhibition of Hsp90-mutant p53 complex formation. The treatment with A452 affected the HDAC6 expression, that was inversely associated with p53 acetylation at lysines 381/382, related with p53 functional activation. A452 treatment blocked HDAC6 nuclear localization, resulting in increased levels of acetylated p53 at Lys381/382. A452 interfered with the HDAC6-Hsp90 chaperone machinery through acetylation and degradation of Hsp90 [49].

In addition, HDAC6 is also involved in the signaling pathway of PI3K (phosphoinositide 3-kinase)/AKT (protein kinase B) and mitogen activated protein kinase (MAPK)/ERK (extracellular signal–regulated kinase) [50]. HDAC6 inhibition affects AKT and ERK dephosphorylation, responsible for the inhibition of cell proliferation and the induction of cell death. Furthermore, HDAC6 inhibition triggers the hyperacetylation of HSP90 leading to decreased levels of phosphorylated AKT and ERK [51,52,53].

## 3. Abnormal Expression HDAC in Lymphoproliferative Disease

Many lymphoid malignancies show increased HDAC expression and activity. In lymphoma cells gene deletions and mutations that inactivate or reduce HAT activity are often found. The reduction of acetylation is associated with the proliferation and survival of lymphoma cells, while increased acetylation is associated with cell tumor growth arrest and cell death. HDACs are considered promising targets for cancer therapy because regulate a variety of cell functions that are involved in cell survival, cell-cycle progression, angiogenesis, and immunity. Their activity is not only limited to histones but also to non-histone proteins as signal transducers, transcription factors, and oncoproteins [54,55,56,57]. Class I HDAC (HDAC1–3 and 8) are predominantly upregulated in hematological malignancies and their altered expression in some cancers has a significant prognostic implication. Different studies have reported an overexpression of HDAC6 in primary and cultured myeloma and lymphoma cells [42,58,59] (Table 2).

Mithraprabhu and colleagues characterized the expression pattern of HDACs in multiple myeloma and correlated the expression with patient outcomes. In that study, the expression of HDACs at a transcriptional level was evaluated utilizing both genetically heterogeneous HMCL (human myeloma cell lines) and primary MM (multiple myeloma) cells compared to normal plasma cells. They observed the overexpression of class I HDAC (HDAC1, HDAC2, HDAC3, and HDAC8) in all HMCL tested, while only 2 of the class II (HDAC5 and HDAC10) were overexpressed. Furthermore, the patients with higher levels of HDAC1–3, HDAC4, HDAC6, and HDAC11 transcripts demonstrated a significantly shorter progression-free survival (PFS) [60]. Overexpression of HDAC1, HDAC2, and HDAC6 and of higher acetylation levels of histone H4, with respect to the normal lymphoid tissue, have been reported by immunohistochemical studies conducted in patients with peripheral T-cell lymphoma (PTCL), and diffuse B large cell lymphoma (DLBCL) [61]. The study conducted by Marquard and collaborators in cutaneous T-cell lymphoma (CTCL) patients reported a high expression level of HDAC1, HDAC2, and HDAC6, and an association between the levels of HDAC2 and histone H4 acetylation and tumor aggressiveness. [59]. The high expression of HDAC has been confirmed in the study conducted by Wang et al. in chronic lymphocytic leukemia (CLL) patients with a significant increase in HDAC of class I including HDAC1 and HDAC3, class II including HADC6, HDAC7, HDAC9, and HDAC10, and class III including SIRT1 and SIRT6 [62]. Additionally, the expression of class I and class II HDACs has been studied in a panel of cell lines and tissue sections from primary lymphoid tumors by Gloghini and colleagues. This study revealed that class I enzymes were highly expressed in all non-Hodgkin lymphoma (NHL) and Hodgkin lymphoma (HL) cell lines and primary tumors studied, including the non-malignant reactive cells in the HL microenvironment. Instead the class II enzyme HDAC6 was variably expressed in different types of lymphoid cell lines compared with HDACs 5, 8, and 10. This variable expression of HDAC6 was not evident in the primary lymphoma sections. Only 4% of primary diffuse large B cell lymphomas (DLBCL) and 18% of HL cases demonstrated detectable levels of HDAC6 [63]. Since HDAC6 is rarely expressed in primary lymphoma cases, Gloghini et al. asserted that it may not be an important therapeutic target in selected lymphoid malignancies. It is important to know that in this study are included only few cases of follicular lymphoma, mantle cell lymphoma, T-cell lymphoma, and plasmacytomas and the expression pattern of HDAC6 in these histological subgroup remains undetermined. The expression of HDAC1, 2, and 3 were studied also in 283 HL and Reed–Sternberg cells (HRSC) on tissue microarray by Adams H. et al. All the HL that was possible to analyze expressed the HDAC2 (*n* = 194) and HDAC3 (*n* = 207) isoforms in mostly 100% of HRSC and tumor-infiltrating lymphocytes. HDAC1 was expressed in 169 of 179 analyzable HL in a mean 82% of HRSC and 172 out of 179 analyzable cases in a mean of 83% of tumor-infiltrating lymphocytes [64].

In the DLBCL and the follicular lymphomas the molecular mechanism leading to lymphoid oncogenic transformation is mediated by overexpression of the transcriptional repressor B-cell lymphoma 6 (BCL6) [66] that is associated with aberrant transcriptional repression through recruitment of HDACs both of class I and class II [67].

Yang et al. conducted a study in leukemia patients observing a significant overexpression of HDACs in CLL patients, with heterogeneous expression of HDACs in myeloid malignancies, such as acute myeloid leukemia and myelodysplastic syndromes, without a leukemia-specific HDAC gene expression profiles [68].

## 4. Anticancer Effects of HDAC Inhibitors and Their Roles in Lymphoproliferative Disease

### 4.1. Classification of HDAC Inhibitors: Specific and Non-Specific HDACis

Since HDACs modulate a variety of cellular functions involved in cell survival and in cancer pathological conditions where the HDACs are overexpressed, the discovery of the association between histone acetylation and malignant pathologies has sparked an interest for HDACs and several new drugs have been developed in last years. All the HDACs require a Zn molecule in their active site and are inhibited by the so called pan HDACis [69], except for the class III HDACs [70]. Based on their chemical structure HDACis can be classified in distinct groups: hydroxamates (TSA, vorinostat/SAHA, ricolinostat/Acy-1215, citarinostat/Acy-241, tubacin, tubastatin) aliphatic acid (phenylbutyrate, valporoic acid), benzamides (entinostat); and cyclic tetrapeptides (romidepsin) [71] (Table 3).

HDACis can have a specific inhibitory effect against some subtypes of HDACs (HDAC isoform-selective inhibitors) or non-specific effects, against all types of HDACs (pan-inhibitors).

An important query is if pan-HDAC inhibitors are potentially more effective therapeutic agents than HDAC-selective inhibitors. The non-specificity of currently available HDACis inhibitors results in modulating the acetylation status of a wide range of protein targets, which may cause undesired toxic effects. Selective inhibition may improve the efficacy and decrease the toxicity of pan-HDAC inhibitors observed in the clinic.

Previous studies have demonstrated the ability of HDACis to enhance drug-induced cytotoxicity, that has been related to activation of proapoptotic pathways. To enhance tumor cell chemosensitivity, the HDACis have been used either individually or mostly in combination with other anti-cancer drugs [72,73,74]. HDACis as single agents are effective in hematological disease given their pleiotropic anticancer activities, however a growing number of studies have demonstrated more efficient and tumor specific anticancer activities of HDAC inhibitors in combination with other drugs. Indeed, several preclinical and clinical studies have indicated that HDAC inhibitors potentiate the efficacy of a variety of drugs such as proteasome inhibitors, lenalidomide, pomalidomide, dexamethasone, and venetoclax [75,76,77,78,79,80]. The synergistic effect between pan HDACis and proteasome inhibitors was attributed to the ability of pan HDACis to repress HDAC6-dependent aggresome function [81,82]. The combination of new agents and HDAC inhibitors will help to develop non-chemotherapy-based regimens that will maintain a high cure rate but will also reduce treatment-related toxicity.

### 4.2. Mechanisms of Actions of HDAC Inhibitors

HDAC inhibitors (HDACis) represent a class of targeted anticancer drugs that inhibit histone deacetylases causing an increase of the acetylated level of histone, which in turn upgrade the expression of the silenced regulatory genes in malignant cells [55,83,84]. HDACis treatment demonstrated favorable results in B-cell lymphomas where the pathogenesis is secondary to the deregulation of the BCL6 proto-oncogene. BCL6 is negatively regulated by p300 acetylation, which interferes with its capacity to recruit HDACs. The pharmacological inhibition of HDAC activity in B-cell lymphoma cells induces the accumulation of the inactive acetylated form of BCL6 causing cell cycle arrest and apoptosis [85]. It is therefore possible to hypothesize the use of HDAC inhibitors to control the activity of BCL-6 on gene expression through the modulation of its acetylation or by influencing the accessibility of the transcription factor on specific DNA regions. Similarly, in aggressive B-NHLs, the aberrant expression of c-Myc protein cooperates with HDAC to promote its transcriptional program and neoplastic transformation [86]. c-Myc represents a valid therapy target to block the proliferation of neoplastic cells as its inhibition has been shown to block the proliferation of the neoplastic clone [87]. Then abrogating the transcriptional activity of c-Myc by blocking the HDAC enzymes could represent an alternative strategy to stop the guided lymphomagenesis from c-Myc [87].

**Figure 2:**
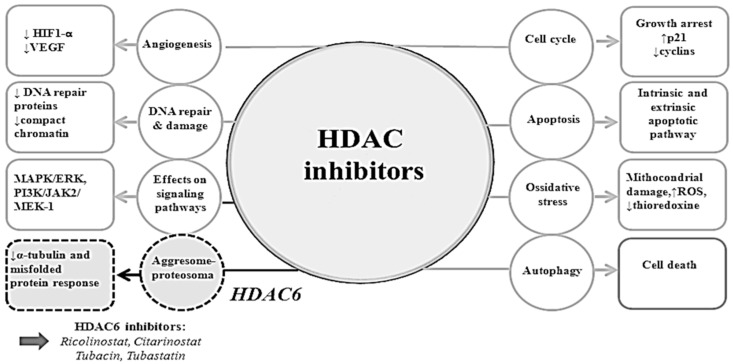
Effects of HDAC inhibitors on tumor cell. Exposure to HDAC inhibitors leads to a wide spectrum of biologic effects, including induction of apoptosis, inhibition of angiogenesis, ossidative stress, effects on signaling pathways, and disruption of the aggresome related to HDAC6 (increases↑; decreases↓).

Mechanisms of anticancer effects of HDACis are not uniform, depending on the type of cancer, on the individual HDAC inhibitor, and its dose, as well as other factor [88]. In cancer cell lines HDACis demonstrated several downstream effects, inducing cell cycle arrest in G1-S phase, activating apoptosis via pro- and antiapoptotic mechanisms of cell death and inhibition of angiogenesis. They also affect the endoplasmic stress response, and are involved in activation or inactivation of tumor suppressor genes or oncogenes controlling cell growth and cell death [89]. Furthemore, HDACis have a pleiotropic effects on signaling pathways that affect proliferation, differentiation, angiogenesis, and cell survival [55,84,90] (Figure 2).

#### 4.2.1. Cell Cycle Arrest

It has been shown that HDACis block cell proliferation and induce apoptosis in hematologic and solid tumor malignancies cell lines causing cell cycle arrest in G1 or G2/M phase, principally by altering the expression of proteins (cyclins, cyclin dependent kinase inhibitors) involved in cell growth [91]. HDACis inhibit the expression of cyclin D [92,93] and cyclin A which reduces the activity of CDK4 and CDK2 leading to cell cycle arrest in G1. HDACis induce upregulation of p21, p27, and p16 leading to inhibition of cell cycle progression after binding to and inactivating CDK4 and CDK2 [94]. The increase of p21 expression is considered to be one of the most important mechanisms leading to HDAC inhibitor mediated G1/S arrest [95,96,97,98].

#### 4.2.2. Apoptosis

It has been demonstrated that HDACis induce apoptosis in tumor cells by regulation of expression of proapoptotic and antiapoptotic genes [99]. The apoptosis induced by different HDAC inhibitors include the activation of both extrinsic and intrinsic pathways which influence death receptors and their ligands [100]. HDACis activate the intrinsic pathway via upregulation of a number of proapoptotic BH3-only *Bcl-2* family genes including Bim, Bid, and Bmf and decrease the expression of the antiapoptotic proteins Bcl-2 family [101]. HDACis are known to activate caspases by mitochondrial or death receptor-mediated pathways [84]. The mechanism involved in the HDACis induced cell death is still unclear, although, oxidative stress has been identified as a mechanism involved in the cytotoxicity of HDACi.

There are different studies showing that HDACis induce ROS (reactive oxygen species) production and caspase activation [102]. Generation of ROS is another key event in HDACis induced cell death, causing DNA damage. ROS production, induced by HDACis, leads to activation of caspase and generates apoptosis in various types of cancer cells [102]. Irregular ROS production can also promote the conformational changes of members of the pro-apoptotic Bcl-2 family increasing the permeability of the mitochondrial membrane. ROS production induced by HDACis was associated to decreased expression of thioredoxin (Trx), a ubiquitous protein with pleiotropic effects that functions as an intracellular antioxidant. Trx stimulates tumor growth and inhibits both spontaneous and drug-induced apoptosis [103,104].

#### 4.2.3. Autophagy

One possible mechanisms of non-apoptotic cell death induced by HDAC inhibitors is induction of autophagy. The importance of acetylation in autophagy control has emerged in the last few years [105,106]. In several cancer models, it has been reported that HDAC inhibitor treatment can cause caspase-independent autophagic cell death. Cell death by autophagy is induced by the conversion of unconjugated microtubule linked protein light chain 3 (LC3-I) to conjugated light chain 3 (LC3-II), the transfer of LC3 to autophagosomes, the increase of acidic vesicular organelles and protein expression associated with autophagy as well as the *Atg5* gene related to autophagy [107,108,109].

#### 4.2.4. Angiogenesis

The inhibition of HDAC can also block cell growth through the inhibition of angiogenesis. Indeed, the HDACs inhibitors are able to increase the acetylation of the pro-angiogenic factor HIF-1α determining its degradation and reducing the expression of the factor VEGF and angiopoietin [110].

#### 4.2.5. Migration

The inhibitors of the HDACs are able to interfere with the downregulating process of metastasis the CXCR4 chemokine receptor, important for homing medullary progenitors and circulating endothelial cells, and prometastatic factors as the metal proteinase MMP2. In particular, through inhibition of HDAC6, HDAC inhibitors reduce microtubule acetylation preventing the migration of cancer cells [111].

#### 4.2.6. Protein–Protein Interactions

HDACs are involved in multiple cellular processes that include protein stability, protein–protein interactions. An important mechanism of the anticancer effect of HDACis is the regulation of cell differentiation by activation of the mitogen-activated protein kinase (MAPK) pathway [112,113]. HDACis also promote acetylation of cytoplasmic proteins, altering function of several oncogenic proteins [55,65,114,115,116].

In addition to the transcriptional effects, HDACis are also involved in acetylation status of non-histone proteins implicated in critical regulatory processes as transcription factors (p53, c-Myc), α-tubulin, hypoxia-inducible factor 1 alpha (HIF-1α), chaperons (HSP90), signaling mediators (STAT3), β-catenin, and many others [54,117]. Finally, It has been reported that HDACis disturb proteasome function and post-transcriptional protein modifications via mechanism that are undefined but may include interference with the aggresome function that is responsible for the deposition of excess protein destined for proteasomal degradation [42]. The expression of HDAC6, a microtubule-associated deacetylase that interacts with polyubiquitinated proteins, is sufficient to rescue degeneration associated with ubiquitin-proteasome system (UPS) dysfunction in vivo in an autophagy-dependent manner [118,119,120]. HDAC6 provides an essential mechanistic link in the compensatory interaction of induced autophagy when the UPS is impaired (Figure 1 and Figure 2).

### 4.3. Pan-HDAC Inhibitors in Lymphoproliferative Disorders: Preclinical and Clinical Data

Pan-HDAC inhibitors can activate several antitumor pathways with potential therapeutic advantages compared to HDAC isotype-specific inhibitors. HDACis are known for their cytotoxicity that discriminate between normal and tumor cells [121] although it remains unclear why tumor cells are more sensitive to HDACi-induced cell death than normal cells. Emerging data suggest that the effects of HDACis in tumor cells may not be only depend on the specificity and selectivity of the HDACis but also on the expression patterns of HDAC enzymes in tumor tissue. The antitumor activity of HDACis has been confirmed in several preclinical and clinical trials for different type of tumors including lymphoid malignancies [122].

Several pan-HDACis have been tested in clinical trials for the treatment of myeloma multiple (MM), follicular lymphoma (FL), Hodgkin’s lymphoma (HL), cutaneous T-cell lymphoma (CTCL), and Diffuse large B cell Lymphoma (DLBCL), and ongoing clinical trials are testing HDACis alone or in combination with other cancer therapeutics for the treatment of B- and T-cell malignancies (Table 3).

So far four HDACis have been approved by the US Food and Drug Administration (FDA) for clinical use. The first two FDA-approved inhibitors are the pan-HDACi, vorinostat (SAHA) [123], which has been approved for the treatments of CTCL and is available as an oral medication, and the class I HDACi romidepsin (istodax, a bacterial cyclic peptide), which is administered intravenously and has been granted FDA approval for CTCL treatment and for peripheral T cell lymphoma (PTCL) (Table 3). HDACis treatment is especially effective in the treatment of CTCL, with favorable response rates of 45% with romidepsin [124]. Romidepsin also induced complete and durable responses in patients with relapsed or refractory peripheral T cell lymphoma across all major PTCL subtypes with objective response rate of 25%, which led to the approval of single agent romidepsin for the treatment of relapsed or refractory PTCL in the US [125]. According to clinicaltrials.gov, romidepsin is currently evaluated in several studies, either as a single agent or in combination with other drugs mainly for treatment of T-cell lymphoma. Belinostat (hydroxamate) was also approved in 2014 for relapsed and refractory PTCL [126]. Finally, panobinostat, another hydroxamate, has been approved by the FDA for refractory multiple myeloma [127]. However, like any anticancer agents, HDACis are also associated with toxicities. The most common grade 3 and 4 adverse events observed with the use of HDAC inhibitors were thrombocytopenia, neutropenia, anemia, fatigue, and diarrhea [125,128,129,130]. In two independent phase 2 trials romidepsin showed an overall response rate of 34% with a durable response of 13–15 months in patients with refractory CTCL and the most frequent toxicities of romidepsin included nausea, vomiting, fatigue, and myelosuppression [131]. In some cases, the HDAC-induced thrombocytopenia can be rapidly reversible upon withdrawal of the drug [71,132].

### 4.4. Selective HDAC6 Inhibitors in Lymphoproliferative Diseases: Pre-Clinical and Clinical Data

The pan-HDAC inhibitors toxicity might be due to their lack of specificity, reducing their tolerability profile. In order to preserve the anticancer effect of HDACis while reducing the toxicity, more selective compounds have been developed. The class IIb, HDAC6, has emerged as one potential selective HDACi. Considering the implication of HDAC6 in cancer progression, this isoenzyme represents a good pharmacological target for selective inhibition potentially reducing the toxicity related to the off-target effects of pan-HDAC inhibitors. It is known that HDAC6 serve as a molecular chaperone, it plays a role in regulating the aggresome function [133,134], and affects the acetylation status of several proteins, including alpha-tubulin, that is important for the regulation of microtubule stability and function. In hematological malignancies, HDAC6 has been reported to be overexpressed in both B- and T-cell lymphomas [61] and its inhibition has demonstrated activity in preclinical models of lymphomas and MM [135,136]. Different HDAC6 selective inhibitors have been synthesized, and most of them belong to the class of hydroxamic acids [137,138] (Table 4). Small molecules such as tubacin and tubastatin have been developed to target HDAC6 [42,139].

#### 4.4.1. Tubacin

This molecule induces microtubule stabilization affecting cell mobility through α-tubulin acetylation [140]. The inhibition of HDAC6 by tubacin results in the increase of Ku70 acetylation, and the secondary release and activation of the proapoptotic protein BAX. The proteasomal degradation of the antiapoptotic protein FLIP promotes the apoptosis [141,142]. The effects of tubacin has been studied in MM and lymphoma. In MM cells, the result of HDAC6 inhibition by tubacin caused acetylation of α-tubulin, induced apoptosis, decreased cell mobility, and inhibited the interaction of HDAC6 with dynein, with a marked accumulation of ubiquitinated proteins [42]. Tubacin was also combined with the proteasome inhibitor bortezomib. The combination of tubacin with bortezomib induced synergistic antitumor activity in MM cells and primary bone marrow plasma cells, and with a cytotoxicity mediated by c-Jun NH2-terminal kinase/caspase activation [42]. In lymphoma cells, the overexpression of HDAC6 in primary lymphocytes and T cell lines increase cell migration in response to cytokines. Knockdown of HDAC6 in T cells decreased chemotactic mobility independently by its enzymatic activity [143]. In Burkitt’s lymphoma cell lines the inhibition of HDAC6 activity by tubacin compromised the migration and invasion of the cells suppressing SDF-1α (stromal cell-derived factor 1) [144]. Although the preclinical data obtained with tubacin were promising, unfortunately the high lipophilicity of this compound did not allow clinical investigations. Therefore, tubastatin A, a tubacin derivative, was synthetized.

#### 4.4.2. Tubastatin

In preclinical studies in myeloma cell lines, tubastatin A synergistically enhanced both bortezomib and carfilzomib-induced cytotoxicity [42,157]. Tubastatin A induced apoptosis and suppressed lymphoma cell colony formation [157]. In preclinical studies in lymphoma cell, Lwin and colleagues, revealed that tubastatin A induced apoptosis and inhibited clonogenic growth of lymphoma cells both in the absence and presence of stroma adhesion, further supporting the role of HDAC6 in cell adhesion-mediated clonogenicity [158]. Despite the positive results in the preclinical setting, this compound demonstrated reduced bioavailability as well.

Both tubacin and tubastatin are not optimized for oral delivery and they have not been tested in clinical trials.

Among HDAC6 inhibitors, only ricolinostat (ACY-1215, rocilinostat) is currently evaluated in clinical trials [122].

#### 4.4.3. Ricolinostat

This compound is a class IIb tubulin deacetylase inhibitor and is the first HDAC6 selective inhibitor that showed promising results in pre-clinical testing and in clinical trials. Ricolinostat is 10–15-fold selective for HDAC6, and 12-, 10-, and 11-fold less active against HDAC1, HDAC2, and HDAC3 (class I HDACs). It has demonstrated in vitro and in vivo activity in MM and lymphoma models, both as a single agent and in combination with other drugs [135,136].

While very well tolerated in the clinic, activity as a single agent however has been limited, and combination strategies have proven more efficacious thus far. Combinations of ricolinostat with lenalidomide, pomalidomide, and bortezomib are currently in clinical study for patients affected by MM [159,160,161,162]. In myeloma cell lines, ricolinostat alone inhibited cell growth, induced α-tubulin acetylation and cell death by apoptosis [135,145]. Synergistic activity has been observed in vitro study, when ricolinostat was used in combination with the proteasome inhibitors bortezomib and carfilzomib. The blockade of both the proteasome and aggresome pathways via combination therapy with the proteasome inhibitors and ricolinostat has synergistic antitumor activity in MM [163]. In myeloma cell lines, low doses of ricolinostat combined with bortezomib triggered synergistic effect resulting in prolonged endoplasmic reticulum stress and apoptosis via activation of caspases [146,148]. The efficacy of the combination was also confirmed in vivo, in two different xenograft SCID mouse models [135].

Recently an in vitro study showed that ricolinostat induced upregulation of CD38 expression on myeloma cells and had a synergistic effect in combination with daratumumab, an anti-CD38 antibody [164].

Experimental results also suggested that ricolinostat has potential efficacy in combination with proven immunotherapeutic drugs such as immune checkpoint inhibitor and monoclonal antibodies [75,162].

Results from clinical studies in myeloma, demonstrated that ricolinostat is quickly absorbed with a half-life of ~3 h [165]. Concentration of ricolinostat increased in a dose dependent manner from 40 to 160 mg, stabilizing at doses ≥160mg. Administration of ricolinostat (40–240 mg once daily or 160 mg twice daily) with bortezomib (1.3 mg/m^2^) or lenalidomide (25 mg) did not affect the pharmacokinetics of the single drugs. Vogl and colleagues conducted a study of ricolinostat in combination with bortezomib and dexamethasone in patients with relapsed or refractory multiple myeloma [147]. Combination therapy with bortezomib and dexamethasone was well-tolerated during dose escalation but led to dose-limiting diarrhea in an expansion cohort of ricolinostat at the dose of 160 mg twice daily. Combination therapy of ricolinostat at the dose of 160 mg daily in a second expansion cohort was well tolerated, with less severe hematologic, gastrointestinal, and constitutional toxicities compared with published data on nonselective HDAC inhibitors. The overall response rate of daily ricolinostat at ≥160 mg, in combination, was 37% and 14% among bortezomib-refractory patients. Samples taken during therapy showed dose-dependent increases of acetylated tubulin in peripheral blood lymphocytes. The combination of ricolinostat with bortezomib overcomes bortezomib resistance in relapsed MM, with a favorable safety profile that offers potential advantages compared with nonselective HDAC inhibition [147]. Ricolinostat has been also studied in combination with the immunomodulator pomalidomide. When 4 mg of pomalidomide and 160 mg once daily or twice daily of ricolinostat were administered, Cmax of ricolinostat was reached ≈1 h after the first daily dose and then decreased to background levels within 6 h [165]. Pharmacodynamic analyses have demonstrated that the mean fold increase in acetylated tubulin is greater than for acetylated histones, indicating selective HDAC6 inhibition [78,160,166]. Ricolinostat has also been studied by Yee and colleagues in combination with lenalidomide and dexamethasone in relapsed refractory myeloma patients [78].

In this study ricolinostat has been administered once daily at the dose of 60 mg on days 1–21 of a 28 day cycle, in combination with 25 mg of lenalidomide and 40 mg of dexamethasone. The most common adverse events were fatigue and diarrhea. Pharmacodynamic studies indicated that ricolinostat, at clinically relevant doses, inhibits HDAC6 while keeping a low and tolerable level of class I HDAC inhibition [78].

Antitumor activity of ricolinostat alone and in combination has also been studied in lymphomas. Amengual et al. demonstrated that targeting HDAC6 with ricolinostat in a panel of 16 lymphoma cell lines (DLBCL, MCL, T-cell lymphoma) inhibited HDAC6 activity and sequestration of misfolded proteins by disrupting transport to the aggresome through acetylation of α-tubulin. This effect activated the UPR-apoptosis pathway shifting the cells towards death. These results were confirmed in a xenograft mouse model of DLBCL [136]. Recently, a study conducted by the same authors utilizing a panel of cell lines and primary patient samples of lymphoma, showed the synergistic effect between ricolinostat and ibrutinib, confirmed in and in vivo xenograft mouse model of DLBCL [149]. Furthermore, the inhibition of HDAC6 leads to upregulation of the IRE1 pathway of the UPR. This in turn was connected with upregulation of the B-cell receptor pathway. The confirmation of the interaction between the UPR and the BCR pathway was further established by demonstrating synergy between ricolinostat and ibrutinib across a panel of cell lines (ABC-DLBCL and MCL) known to be sensitive to BTK inhibitors [149].

Interactions between ricolinostat and carfilzomib were examined in non-Hodgkin lymphoma (NHL) models, including DLBCL and MCL [148]. In vitro, ricolinostat works synergistically with carfilzomib in multiple DLBCL and MCL systems, including bortezomib-resistant cells. The results indicate that drugs combination induced cell death through multiple stress-related mechanisms accompanied by increases in DNA damage (γH2A.X), G2–M arrest, and the marked induction of mitochondrial injury. Combination treatment with carfilzomib and ricolinostat increased concentration of reactive oxygen species (ROS). In an MCL xenograft model the treatment with carfilzomib and ricolinostat was well tolerated, suppressing tumor growth and increased animals survival [148].

In a preclinical study conducted recently by our group [151] we tested the combination of ricolinostat with bendamustine in lymphoma cell lines showing synergistic apoptosis-inducing effects which is mediated by a corresponding effect on microtubule stabilization. The synergistic effect was accompanied with the increased ROS, activation of caspase and modulated by Bcl-2 proteins family. Exposure to ricolinostat induced the acetylation level of α-tubulin, the extend of which was not further modified by bendamustine [151]. This demonstrates that HDAC6 plays an oncogenic role in DLBCL via indirect activation of MET signaling.

A recent study in lymphoma, showed that HDAC6 plays an oncogenic role in DLBCL and the combination of ricolinostat with crizotinib (ALK inhibitor) generated a strong synergistic effects. In vivo efficacy of drug combination has been evaluated using a human DLBCL xenograft mouse model. Ricolinostat (50 mg/kg) and crizotinib (50 mg/kg) in combination treatment significantly inhibited tumor growth by ~87% [150].

A second HDAC6 inhibitor, derived from ricolinostat, is citarinostat (ACY-241), that is structurally similar to ricolinostat but administered as a tablet rather than an oral solution.

#### 4.4.4. Citarinostat

This agent is a second generation HDAC6 selective inhibitor with 13 to 18-fold selectivity towards HDAC6 in comparison to HDAC1-3 [167]. Bae et al. demonstrated mechanisms whereby citarinostat in a dose- and time-dependent fashion augments immune response and mediates anti-MM activity both by decreasing CD138^+^ tumor cells and tumor-promoting immune cells and their expression of immune checkpoints, as well as by promoting the activation of antigen specific CD8^+^ T cells [168]. Ray and colleagues examined the combination of citarinostat with anti-PDL-1 antibody. For these studies the authors used a co-culture model of immune effector cells (plasmacytoid dendritic cells, T cells, and NK cell) and MM cells. Combination treatment triggered a more robust NK-cell mediated cytolytic activity against MM cell than each agent alone [152]. Citarinostat showed efficacy in combination with the immunomodulators lenalidomide and pomalidomide [80]. At the molecular level, combination treatment resulted in increased apoptosis as well as cell cycle arrest, coupled with decreased expression of pro-survival factors survivin, Myc, and IRF4. This combination effect in vitro translated to significant efficacy at reducing tumor growth in an in vivo MM xenograft model [80]. These results support the rationale of the Phase 1a/b clinical trial (NCT02400242) [122] exploring combination treatment of citarinostat plus pomalidomide and dexamethasone in MM patients [80,122,169]. In addition, preliminary results of a preclinical study conducted recently by our group indicated that citarinostat can be potentially combined with momelotinib, a JAK2/STAT3 inhibitor in lymphoid malignancies [170]. In this study the antitumor activity of citarinostat/momelotinib combination is mediated by the pro-apoptotic effect and by downregulation of JAK2/STAT3 pathways and its downstream mediators [170]. Both, ricolinostat and citarinostat are evaluated as monotherapy and in combination in phase 1 and 2 clinical trials in patients with multiple myeloma (NCT01997840, NCT02400242) and lymphoid malignancies (NCT02091063) [122].

## 5. Future Developments of HDAC Inhibitors

Cancer drug research is a rapid expanding field, with new innovative products in active development. Scientists are making a lot of progress in understanding how changes in DNA within normal cells can cause them to turn into malignant cells. Advances in understanding DNA changes in lymphoma cells have already led to improvements and highly effective drugs in these neoplasms. Ever since HDAC inhibitors were found active in various clinical trials, the efforts to discover more efficient and selective HDAC inhibitors have been continually intensified.

Recently, the AK-DACi (a first-in-class alkylating deacetylase inhibiting molecule) tinostamustine (EDO-S101) has been developed by Mundipharma EDO GmbH. It is a new drug that chemically combines an alkylating agent with a pan-histone deacetylase inhibitor to simultaneously damage DNA and block damage repairs [171]. The molecule was designed to maintain the fully functional capacity of both compounds in order to increase the efficiency of both mechanisms decreasing toxicity and providing more convenient administration. The rationale for using tinostamustine arises from the hypothesis that histone acetylation induced by the novel radical would result in a more open chromatin structure which would be particularly susceptible to the alkylating effect of bendamustine. Compared to the single drugs, the combined function in one molecule showed superior activity. Preclinical studies showed that the apoptosis induced by tinostamustine occurred at substantially low concentrations following a very strong DNA damage response after exposure in vitro and in vivo. Indeed, tinostamustine showed a strong preclinical activity in vivo against multiple myeloma (MM), leukemia, and B-cell lymphomas with a toxicity profile similar to bendamustine [172]. López-Iglesias and colleagues demonstrated that tinostamustine has a potent activity in MM cell lines and ex vivo in cells isolated from MM patients, which was higher than that of bendamustine and this activity was confirmed in vivo, in a CB17-SCID murine plasmacytoma model and in de novo Vk*MYC mice, leading to a significant survival improvement in both models [173]. Furthermore, studies in vitro in MM cell lines, conducted by Besses et al. revealed that tinostamustine, is a potent ER stress-inducing, HDAC6-inhibiting, immunomodulatory, cell cycle inhibiting, pro-apoptotic, and c-MYC-antagonistic activity, in contrast to vorinostat or bendamustine [174]. In addition, tinostamustine exhibits strong synergy when given as part of combination therapy with agents such as bortezomib and dexamethasone and may enhance the efficacy, offering the possibility of improved duration and depth of response [174,175]. The first clinical study with tinostamustine is ongoing in patients with haematological malignancies (clinicaltrials.gov identifier: NCT02576496) [122].

Recently, Karus Therapeutics Ltd. has designed and developed a novel class of highly-selective inhibitors of HDAC6 with potential antineoplastic activity which combine the effectiveness of targeted therapy and immunotherapy [176]. A HDAC6-specific inhibitor called KA2507, inhibits tumor growth through regulation of aggresome formation, and inhibition of PD-L1 expression via decrease of STAT3 phosphorylation [176]. Inhibition of HDAC6 confers a cancer immunotherapeutic response by regulating immune checkpoint markers within the tumor microenvironment. Clinical trial is conducted by Karus Therapeutics in patients with PD-L1 expressing solid tumors. (clinicaltrials.gov identifier: NCT03008018) [122].

## 6. Conclusions

Over the past few decades, the growing understanding of epigenetics has led to a rapid expansion of knowledge by researches on its role in cancer development. HDAC inhibitors represent an encouraging class of antitumor drugs given the series of molecular and biological responses that these agents can produce and minimal toxicity to normal cell. The therapeutic efficacy of the HDAC inhibitors has been seen primarily in hematological malignancies with four of them approved for cutaneous and peripheral T-cell lymphoma. Their use in combination with other agents reveal a more useful application. Pan-HDAC can activate more antitumor pathways and this may lead toxicities due to their lack of specificity. Furthermore, this limits duration of treatment especially when they are used in combination with other agents with overlapping toxicities.

Recently, the attention has concentrated on development of more selective HDACis based on the premise that such agents may be more tolerable than pan-HDACis. HDAC6 has become a promising target molecule, due to its structure and functions, and for its non-histone substrates which play a central role in malignancies development. The inhibition of HDAC6 is not expected to produce severe toxicity, as documented by the good tolerability of compounds with preferential ability to inhibit this isoform [32,36,135,177,178]. HDAC6 stands out from the other HDACs in deacetylating cytoplasmic proteins, in employing deacetylation-independent effects and in the success that has been obtained in the development of isoform-specific inhibitors of its enzymatic action which have reached clinical trial. Different HDAC6 selective inhibitors have been studied so far. Among the several molecules with HDAC6 selective inhibition properties, ricolinostat and citarinostat emerge as the most promising drugs, and are now under investigation in combination with dexamethasone and bortezomib or an immunomodulatory agents for the treatment of relapsed and refractory MM. Ricolinostat, the first-in-class HDAC6 selective inhibitor, has demonstrated good toxicity profile in patients and preliminary evidence of anti-myeloma efficacy in combination with lenalidomide and dexamethasone [78]. The antitumor activity of ricolinostat alone and in combination has also been confirmed in lymphomas [136]. Citarinostat was designed as a second generation orally available and HDAC6 selective inhibitor with improved solubility properties over the structurally related inhibitor ricolinostat. Both ricolinostat and citarinostat have synergistic anticancer effects with many other antitumor reagents suggesting that combination of this HDAC6 inhibitor and other anticancer drugs can be very attractive therapeutic strategy. Therefore, it would be of interest for future studies to assess the ability of selective HDAC inhibitors, to increase the cure rate of patients with lymphoproliferative disease.

## Figures and Tables

**Table 1:** Histone deacetylases (HDACs) classification, their localization and biological functions.

Class	Members	Cellular Localization	Biological Functions
I	HDAC1	Nucleus	Proliferation control, apoptosis; p21 and p27 CDK (cyclin-dependent kinase) inhibitor repression; Represses transcription; Binds to transcription factors; Resistance to chemotherapy; Suppresses cytokine production in activated T cells and during T effector cell differentiation
	HDAC2	Nucleus	Negatively regulates transcription by being recruited to DNA as a corepressor; Proliferation control; Apoptosis
	HDAC3	Nucleus	Proliferation; Differentiation, represses transcription; Binds to transcription factors; Deacetylates FOXP3 (forkhead box P3) that reduces Treg development and suppressive function
	HDAC8	Nucleus	Proliferation; Differentiation
IIA	HDAC4	Nucleus/Cytoplasm	Differentiation, angiogenesis; Deacetylates BCL6 (B-cell lymphoma 6) which activates genes for lymphocyte activation
	HDAC5	Nucleus/Cytoplasm	Differentiation; Deacetylates BCL6 which activates genes for lymphocyte activation
	HDAC7	Nucleus/Cytoplasm	Angiogenesis; Suppresses Nur77 expression during TCR (T-cell receptor) negative selection; Regulates gene expression during TCR positive selection; Deacetylates BCL6 which activates genes for lymphocyte activation
	HDAC9	Nucleus/Cytoplasm	Deacetylates FOXP3, which reduces Treg development and immunosuppressive activity
IIB	HDAC6	Cytoplasm	Regulation of protein degradation both via aggresome and the regulation of Hsp90 chaperone activity; Migration; Angiogenesis; Controls IgM and IgG levels upon antigen stimulation; T-cell migration; Immune synapse formation; Deacetylates FOXP3 that decreases Treg development and immunosuppressive activity
	HDAC10	Cytoplasm	Angiogenesis
III	SIRT 1	Nucleus, Cytoplasm	DNA repair/genome stability; Chromatin organization; Stress; Cancer
	SIRT 2	Nucleus	Mitosis; DNA repair; Chromatin condensation
	SIRT 3	Mitochondria	Cancer, chromatin silencing; DNA repair; Cellular stress
	SIRT 4	Mitochondria	have not yet been fully determined
	SIRT 5	Mitochondria	have not yet been fully determined
	SIRT6	Nucleus	DNA repair/genome stability; Telomeric chromatin/senescence
	SIRT7	Nucleus	Cellular transformation
IV	HDAC11	Nucleus	Regulates the protein stability of DNA replication factor CDT1 (chromatin licensing and DNA replication factor 1) and the expression of IL-10; Suppresses IL10 expression in APCs (antigen presenting cells)

**Table 2:** Expression of Histone Deacetylases (HDACs) in lymphoproliferative disease.

Class	Members	Expression of HDACs Increased in Lymphoproliferative Disease (Cell Lines and Primary Cell)	Reference
I	HDAC1	MM, HL, MCL, DLBCL, ALCL, CLL PTCL,	[59,60,61,62,63,64]
	HDAC2	MM, HL, MCL, DLBCL, ALCL, PTCL	[59,60,61,63,64]
	HDAC3	MCL, CLL, DLBCL, HL; MM	[59,60,61,62,63,64,65]
	HDAC8	MM	[60]
IIA	HDAC4	DLBCL, PTCL	[61,63]
	HDAC5	MM	[60]
	HDAC7	CLL, MCL	[59,61,62,63]
	HDAC9	CLL, MCL	[59,62,63]
IIB	HDAC6	MM, MCL, DLBCL, PTCL, CTCL, CLL,	[59,60,61,62,63,64]
	HDAC10	CLL, MCL, HL	[59,61,62,63]
III	SIRT 1	CLL	[62]
	SIRT 2		
	SIRT 3		
	SIRT 4		
	SIRT 5		
	SIRT6		
	SIRT7	CLL	[59,62,63]
IV	HDAC11	MCL, HL	[63]

MM = Multiple Myeloma; HL = Hodgkin’s lymphoma; MCL = Mantle Cell Lymphoma; DLCL = Diffuse Large B Cell Lymphoma; ALCL = Anaplastic Cell Lymphoma; CLL = Chronic Lymphocytic Leukemia; PTCL = Peripheral T cell Lymphoma; CTCL = Cutaneous T Cell Lymphoma.

**Table 3:** Histone deacetylase inhibitors (pan and selective) in clinical trials of lymphoproliferative disease.

Class	HDACis	Target HDAC	Clinical Trial Active in Lymphoproliferative Disease (clinicaltrials.gov)
Hidroxamic acids	Trichostatin A	Pan	Preclinical
	Vorinostat/SAHA	Pan	* Phase I/II/III MM and lymphoma
	Belinostat	Pan	** Phase I/II Lymphoma
	Panobinostat	Pan	*** Phase I/II MM and lymphoma
	Givinostat	Pan	Phase I/II completed for MM and lymphoma
	Resminostat	Pan	Phase II CTCL
	Abexinostat	Pan	Phase I/II completed for MM and lymphoma
	Quisinostat	Pan	Phase I/II completed for MM and lymphoma
	Ricolinostat/Acy-1215	II selective	Phase I/II clinical trials for MM and lymphoma
	Citarinostat/Acy-241	II selective	Phase I MM
	Practilinostat	I, II, IV	/
	CHR-3996	I	/
Aliphatic acid	Valproic acid	I, IIa	Phase I/II completed for lymphoma
	Butyric acid	I, IIa	Phase I/II completed for lymphoma
	Phenylbutyric acid	I, IIa	Phase I/II completed for MM and lymphoma
Benzamides	Entinostat	I	Phase I/II completed—MM. Phase I/II—lymphoma
	Tacedinaline	I	Phase II completed—MM.
	4SC202	I	Phase I completed—Advanced Hematologic
			Malignancies
	Mocetinostat	I, IV	Phase I/II clinical trials—lymphoma
Cyclic tetrapepides	Romidepsin	I	Approved for * CTCL and ** PTCL
			Several studies of phase I/II lymphoma
			Phase I/II clinical trials—MM.
Sirtuins inhibitors	Nicotinamide	Class III	Phase I/II MM. Phase I lymphoma
	Sirtinol	SIRT 1 and 2	Preclinical
	Cambinol	SIRT 1 and 2	Preclinical
	Ex-527	SIRT 1 and 2	Preclinical

CTCL = Cutaneous T-cell lymphoma; PTCL = peripheral T-cell lymphoma; MM = Myeloma Multiple * approved by FDA for CTCL; ** approved for PTCL; *** approved for MM.

**Table 4:** HDAC6 inhibitors in lymphoproliferative disease.

HDAC6 Inhibitors	Lymphoproliferative Disease	Preclinical and Clinical Study (Ref.)	Clinical Trials State
Ricolinostat (Acy-1215)	MM cell	Alone [145]	Phase 1/2 combo poma and dex in MM (NCT01997840) (active)
		+ Bortezomib [135]	
		+ Carfilzomib [146]	
		+ Lenalidomide [78]	
		+Dexamethasone [78,147]	
	Non-NHL	+ Carfilzomib [148]	Phase 1/2 combo lena e dex in MM (NCT01583283) (active)
	DLBCL, MCL, TCL	+ Bortezomib [136]	Phase 1 combo poma and low-dose dex in relapsed-and-refractory MM (NCT02189343) (active)
	DLBCL	+ Ibrutinib [149]	Phase 1/2 combo bort and dex in relapsed and refractory MM (NCT01323751) (termined)
		+ Crizotinib [150]	
	FL, MCL, TCL	+ Bendamustine [151]	Phase 1 /2 relapsed or refractory lymphoid malignancies (NCT02091063) (recruiting)
Citarinostat (Acy-241)	MM and MCL	+ Pomalidomide [80]	Phase 1 combo poma and dex in MM (NCT02400242) (active)
		+ Lenalidomide [80]	
	MM	+ anti-PD-L1 [152]	
Tubacin	MM and lymphoma	+ Bortezomib [42,153]	Preclinical studies. Compound not tested in clinical trials: it is not optimized for oral delivery
	Burkitt’s lymphoma	[144,154]	
Tubastatin A	Lymphoma	[155,156]	Preclinical studies compound not tested in clinical trials: It is not optimized for oral delivery
